# The Mechanical Properties Relationship of Radiation-Cured Nanocomposites Based on Acrylates and Cationic Polymerized Epoxies and the Composition of Silane-Modified Tungsten Disulfide Nanoparticles

**DOI:** 10.3390/polym15143061

**Published:** 2023-07-16

**Authors:** Yarden Gercci, Natali Yosef-Tal, Tatyana Bendikov, Hanna Dodiuk, Samuel Kenig, Reshef Tenne

**Affiliations:** 1 Department of Polymer Materials Engineering, Shenkar College, Anna Frank 12, Ramat-Gan 5252626, Israel; yardengercci@gmail.com (Y.G.); natashiyo@gmail.com (N.Y.-T.); hannad@shenkar.ac.il (H.D.); 2 Department of Materials and Interfaces, Weizmann Institute of Science, Rehovot 7610001, Israel; tatyana.bendikov@weizmann.ac.il (T.B.); reshef.tenne@weizmann.ac.il (R.T.)

**Keywords:** tungsten disulfide, nanoparticles, radiation curing, cationic polymerization, acrylate, epoxy

## Abstract

The effect of semiconducting tungsten disulfide (WS_2_) nanoparticles (NPs), functionalized by either methacryloxy, glycidyl, vinyl, or amino silanes, has been studied in photocuring of acrylate and epoxy resins (the latter photocured according to a cationic mechanism). The curing time, degree of curing (DC), thermal effects, and mechanical properties of the radiation-cured resins were investigated. X-ray photoelectron spectroscopy (XPS) and transmission electron microscopy (TEM) analyses confirmed that a silane coating was formed (1–4 nm) on the NPs’ surface having a thickness of 1–4 nm. Fourier transition infrared (FTIR) was used to determine the DC of the nanocomposite resin. The curing time of the epoxy resin, at 345–385 nm wavelength, was 10 to 20 s, while for acrylate, the curing time was 7.5 min, reaching 92% DC in epoxy and 84% in acrylate. The glass transition temperature (Tg) of the photocured acrylates in the presence of WS_2_ NPs increased. In contrast to the acrylate, the epoxy displayed no significant variations of the Tg. It was found that the silane surface treatments enhanced the DC. Significant increases in impact resistance and enhancement in shear adhesion strength were observed when the NPs were treated with vinyl silane. A previous study has shown that the addition of WS_2_ NPs at a concentration of 0.5 wt.% is the optimal loading for improving the resin’s mechanical properties. This study supports these earlier findings not only for the unmodified NPs but also for those functionalized with silane moieties. This study opens new vistas for the photocuring of resins and polymers in general when incorporating WS_2_ NPs.

## 1. Introduction

Radiation curing of thermoset resins offers various advantages, including curing on-demand, low viscosity, good surface adhesion to various substrates, high modulus, good appearance of the final coating, zero volatile organic compounds (VOC), etc. However, radiation-cured thermosets are brittle, and the curing process is limited to low-thickness and transparent formulations [1]. Radiation curing of epoxy is carried out through the cationic mechanism of ring-opening reactions, while acrylate resins are photocured via a radical mechanism [2]. The photocuring process is highly selective due to the specific absorption spectrum of the photo-initiator, which is sensitive only to certain wavelengths [3,4].

WS_2_ nanoparticles (NPs) with fullerene-like structures (IF-WS_2_) and nanotubes were first synthesized in 1992 [5]. They were found suitable for improving the modulus, shear adhesion strength, and fracture toughness simultaneously with increasing the glass transition temperatures of the nano-reinforced polymers upon adding a low percentage of the NPs [6,7,8].

The properties of nanocomposites are affected by the NPs’ concentration, size, shape, and, most importantly, the physio-chemical affinity to the polymer matrix. In general, the optimal concentration of the nanoparticles in the matrix is 0.3–1.0 wt.%, which has been verified through numerous studies [1,2]. Adding larger amounts of nanoparticles induces their agglomeration, which has a deleterious effect on the nanocomposite properties. The interface controls the degree of interaction between the NPs and the matrix. Owing to their high surface area and relatively high surface energy, NPs have the tendency to agglomerate. Therefore, they cannot be easily dispersed in the polymer matrix. To overcome this propensity, the surface of the NPs can be modified by chemical or physical means, thereby improving the interfacial adhesion with the matrix and providing better dispersion. Silanization reactions were found to be successful in modifying the surface of various NPs [9]. The hydrophilic end-group of the silane interacts chemically with the surface of the NPs, while the hydrophobic end interacts with the hydrophobic matrix, which enables their better dispersion. Moreover, the functional group of the silane moiety can form a covalent bond with the matrix and promote the stress transfer from the matrix to the NPs. Prior to this work, a few works described the surface modification of tungsten disulfide NPs by silane moieties. Shneider et al. [10] used acryloxy silane to treat the WS_2_ NPs in epoxy resins. Good dispersion and compatibility were obtained in the epoxy resin. Moreover, a distinct nodular morphology was induced on the fractured surfaces as a result of nucleation by the compatible NPs. In the case where the alkyl silane treatment was used, cavitation morphology was induced following mechanical loading, which is the result of incompatibility with the epoxy resin. The fracture toughness results showed an increase of 70% for nanocomposites containing alkyl-silane-treated WS_2_ NPs compared with the neat epoxy.

Obradović et al. [11] investigated hybrid nanostructures of oxidized carbon nanotubes and silane-treated fullerene-like WS_2_ as reinforcement for Aramid fabric composites. They showed that the nanoreinforcement enhanced the dynamic mechanical and anti-stabbing properties of the composites. The tensile strength increased by more than 50% and the tensile energy by close to 25%, while the impact toughness improved by 48% compared to the conventional Aramid composite.

Haba and coworkers [12] studied the dispersion in ethanol by sonification of IF-WS_2_ modified by three different silanes, Hexyltrichlorosilane (HTCS), 3-glycidoxypropyltrimethoxysilane (GPTMS), and (3-(2-aminoethylamino)propyltrimethoxysilane (AATMS), using X-ray photoelectron spectrometry, infrared spectroscopy, titration, thermogravimetric analysis, and mass spectroscopy. The measurements indicated that the modifications with HTCS and AATMS were successful, while that with GPTMS was not.

Jenson et al. [13] modified WS_2_ NPs successfully by treating them with GPTMS. The surface modification of WS_2_ NPs with GPTMS improved the wettability of the filler materials. FTIR showed that a chemical reaction occurred between the WS_2_ NPs and the GPTMS. Using a silane coupling agent, surface modification of metal particles was used to provide better compatibility with dispersing media to avoid agglomeration of the particles and to impart chemical reactivity to the particles. In another study [14], silane moieties were grafted on WS_2_ NPs, which were oxidized at their surface by ultrasonic treatment in an acidic solution. The alkyl chains of the silane moieties led to excellent dispersion of the nanoparticles in lubricating oils, enhancing their tribological behavior markedly.

In this study, surface modification of WS_2_ NPs was carried out with different types of silanes followed by incorporation of the surface-modified NPs, for the first time, within radiation-cured acrylate and cationic polymerized epoxy resins. The main focus here is placed on the mechanical and thermal properties of the nanocomposite resins compared with the neat resins.

## 2. Experimental Section

### 2.1. Materials

A commercial powder of WS_2_ NPs (MKN-WS2-090) with average size of 90 nm and irregular oval shape was purchased (M K Impex Corp, Mississauga, ON, Canada). Three types of thermosets were investigated here: 1. Acrylate resin photocured in radical reaction. The resin was based on a blend of 70% isobornyl methacrylate (IBMA) and isobornyl acrylate (IBOA), and 30 wt.% urethane diacrylate (Ebecryl 230, Allnex, Wiesbaden, Germany). Furthermore, the resin contained 3-methacryloxypropyltrimethoxy silane (dynasilan MEMO), photo-initiator (irgacure 819, BASF, Ludwigshafen, Germany), and hydrophobic fumed silica NPs (AEROSIL R-972, Piscataway, NJ, USA). 2. The second resin is epoxy cured according to a cationic mechanism. The resin is based on 77% aliphatic epoxy and 23% linear polyester diol (Capa 2043, Ingevity, North Charleston, SC, USA). Furthermore, the resin contained [2-(3,4-epoxycyclohexyl)ethyl]trimethoxy silane (Echtmo), photo-initiator (speedcure 976), and hydrophobic fumed silica NPs (AEROSIL R-972). These resins were supplied by CollTech (Hessen, Germany) and are cured by a light source with 345–385 nm wavelength. 3. The third resin is another epoxy supplied by Polymer G (Gvulot Israel). It consists of EPV 3420TX without thermal additive and will be designated as EPGnTA. The generic formulation of this resin is aliphatic epoxy (45–50 wt.%), methyl acrylate (8–10 wt.%), epoxy acrylate (15–18 wt.%), polyester polyol (15–18 wt.%), fumed nano-silica (4–6 wt.%), and photo-initiator blend of sulfonium-based cationic photo-initiator (PI) and radical PI (3–5 wt.%). These resins were cured in 395 nm wavelength irradiation.

For the surface functionalization of the WS_2_ nanoparticles (NPs), we have used four types of silanes: 3-(methacryloyloxy) propyltrimethoxysilane, (3-glycidyloxypropyl) trimethoxysilane, vinyltrimethoxysilane, and 3-aminopropy(triethoxy) silane.

### 2.2. Silane Functionalization

Mixture of ethanol, distilled water, and silane in a ratio of (95:5):1 was first prepared. The pH of the solution was reduced to 4.5 dropwise with acetic acid. The solution was then mixed with a magnet stirrer for 1 h. To this solution, 1.5% of WS_2_ NPs (after vacuum annealing at 70 °C for 2 h) were added. The solution with the NPs was mixed in a bath sonicated (Ultrasonic, TRANSSONIC TS 540, Elma, Germany) for 2 h. The coated WS_2_ NPs were rinsed with fresh ethanol three times and dried under vacuum for 2 h at 120 °C [15].

The quality of the particle coating was analyzed by FTIR (ATR-FTIR model Bruker Alpha-T, Billerica, MA, USA). X-ray photoelectron spectroscopy (XPS) measurements were carried out with Kratos AXIS ULTRA system using a monochromatic Al Kα X-ray source (hν = 1486.6 eV) at 75 W and detection pass energies ranging between 20 and 80 eV. Wide-scan spectra were taken with the pass energy of 80 eV, while high-resolution spectra of the elements were taken with the pass energy of 40 eV. The survey spectra were measured with a step size of 100 meV, while high-resolution spectra of the elements were measured with a step size of 25 meV. In general, the binding energy of the instrument was calibrated using standard procedure, i.e., measurement of the spectra of Au 4f, Ag 3d, and Cu 2p cleaned standards and fitting the position of the Fermi level- E_F_ to 0 eV. Curve fitting analysis of the W4f spectra was based on linear or Shirley background subtraction and application of Gaussian–Lorenzian line shapes. One has to recall, though, that the entire work is based on solution chemistry at ambient conditions, which may lead to some surface contamination. Therefore, the XPS analysis cannot be directly related to that of similar materials handled and processed under ultra-high vacuum conditions [16,17]. On the other hand, being 90 nm in size and consisting of >10 walls, the WS_2_ NPs are less prone to oxidation and surface-induced reactions compared to a monolayer or a few layers of WS_2_. The morphology of the NPs’ powders was characterized by high-resolution scanning electron microscope (HR-SEM) (Zeiss Supera 55, Zeiss, Oberkochen, Germany), using secondary electrons (SE) detector at 10 kV. Energy dispersive X-ray spectroscopy (EDS) analysis and mapping were performed using a retractable four quadrants detector (Bruker QUANTAX FlatQUAD), which was installed on a Zeiss Ultra 55 SEM. The quantification of the elements is based on standard and self-calibrating spectrum analysis procedure, using the ZAF matrix correction formulas. High-resolution transmission electron microscopy (HR-TEM) analysis was performed with a field-emission gun operating at 200 kV (ThermoFisher model Talos™ F200S TEM).

### 2.3. Dispersion and Distribution of the Nanoparticles

The dispersion of the surface-modified WS_2_ NPs in different resin matrices was carried out with microtip sonication (Q700, Qsonica L.L.C, Newtown, CT, USA) and a vortex mixer at 2400–3000 rpm (Wizard IR Infrared Vortex Mixer, VELP Scientifica, Usmate, Italy). The mixing of the composite resins was carried out for an hour to achieve a homogenous dispersion of the NPs. First, the uniformity of the film and the distribution of the NPs were examined by an optical microscope (Axioplan-ZEISS, Oberkochen, Germany) with ×200 magnification.

To examine the dispersion of the NPs on a microscopic scale, EDS analysis was performed on a fractured surface of the resin. For this purpose, a specimen of the epoxy EPGnTA (#3) with 0.5 wt.% WS_2_ NPs functionalized with vinyl silane was used. The film was dipped into liquid N_2_ Dewar for 10 min and subsequently fractured mechanically. A thin (4 nm) iridium coating was evaporated on the sample to increase the surface conductivity of the film.

### 2.4. Curing

Attenuated total reflectance Fourier transform infrared spectroscopy (ATR-FTIR) (Bruker model Alpha-T, Billerica, MA, USA) was employed to validate the effect of the WS_2_ NPs (silane-coated and uncoated) on the curing kinetics of commercially available radical and cationic photo-cured resins. Wavenumber frequencies ranged from 375 cm^−1^ to 4000 cm^−1^.

The degree of conversion (DC) was calculated by using Equation (1).

(1) $$\mathrm{DC}\;=\;100\;\times \;\;\left(1\;-\;\frac{\mathrm{normalized}\;\mathrm{polymer}\;}{\mathrm{normalized}\;\mathrm{monomer}\;}\right)$$

#### Degree of Conversion Calculation

The data of the acrylate (#1) was normalized with respect to the C=O peak at 1660–1760 cm^−1^. The carbonyl peak is located at 1620–1650 cm^−1^. The data of epoxy (#2) was normalized with respect to the C-H group peak at 2960–2825 cm^−1^. The oxirane peak is located at 794–780 cm^−1^. The data of EPGnTA (#3) was normalized with respect to the alkyl group peak at 2820–2960 cm^−1^. The oxirane peak is located at 770–830 cm^−1^. The DC accuracy was evaluated at 0.5% to 1%.

### 2.5. Photocuring Kinetics

Sample’s dimensions for all three resins were 25 × 6 × 0.3−0.4 mm^3^. The following samples were prepared and measured for each of the three resins: 0 wt.% (neat resin) and 0.5 wt.% WS_2_ NPs, both coated and uncoated. To study the curing kinetics of the acrylate (#1), the curing times were measured after 3, 4, 5, 7.5, and 10 min. For the epoxy (#2) and EPGnTA (#3), the samples were measured after 10, 20, 30, 40, 60, and 120 s.

### 2.6. Dynamic Mechanical Analysis (DMA)

A dynamic mechanical analysis (DMA Q800, TA Instruments, New Castle, DE, USA) was performed in order to determine the transition temperatures and examine the effect of the added WS_2_ NPs on the thermal properties of the two epoxy resins and the acrylate. The following samples were prepared and measured for all three resins: 0 wt.%, 0.5 wt.% coated, and uncoated WS_2_ NPs. The samples for the DMA analysis were cured for acrylate (#1) 7.5 min and epoxy (#2) and EPGnTA (#3) 4 min. The sample’s dimensions were 25 × 6 × 0.3−0.4 mm^3^.

### 2.7. Shear Adhesion Analysis

Single lap shear adhesion tests were performed in order to measure the shear strength. The single-lap shear measurements were carried out according to ASTM D1002 (Instron 4481, Grove City, PA, USA). The thickness of the layer was 0.1–0.2 mm. The resin was applied and cured between two 2 mm thick glass fiber-reinforced epoxy sheets (FR4). The joint lap length was 12.6–13 mm, and the FR4 width was 25 mm. The FR4 sheets were mechanically polished to increase their surface roughness and improve adhesion. Samples were placed ~4 cm from the LED source. The specimens with acrylate resin (#1) were cured for 18 min at 345–385 nm. The samples with epoxy resin (#2) were cured for 10 min at 345–385 nm. The specimen with EPGnTA resin (#3) was cured for 18 min at 395 nm.

### 2.8. Impact Strength

The resistance to impact loading of the material was conducted for the EPGnTA (#3) resin only. Samples were measured using a modified ASTM D-256 IZOD impact mode with a 0.5 J pendulum (Resil 5,5, Ceast, Milano, Italy). The thickness of the samples was 0.3–0.5 mm, and they were different from the standard ASTM specimen (3–12.7 mm). In order to compare the data for the different specimens, the percentage of improvement obtained between the various compositions is reported in addition to the absolute values of the impact strength. The fractured surfaces were examined using SEM (JSM-IT200, Jeol, Tokyo, Japan). Samples were coated with a few nm thick gold film (SC7620, Quorum Technologies Ltd., East Sussex, UK).

## 3. Results and Discussions

### 3.1. Characterization of the Surface of Treated WS_2_ Nanoparticles

The typical IR-spectrum of WS_2_ includes an 870 cm^−1^ peak typical of the S-S bond and another peak characteristic for the W-S bond at 570–625 cm^−1^ wavenumbers [18]. After the surface modification, the WS_2_ NPs were first characterized by ATR-IR. In the resulting IR spectrum, changes were observed in the IR spectra of the WS_2_ NPs before and after the silane surface modification. New peaks related to silane appeared for all types of treatments. The peak in 1189 cm^−1^ is assigned to the Si-O-CH_3_ vibration [19], while the peak in 1070–1104 cm^−1^ is characteristic of the vibration of the Si-O-C group [20].

For WS_2_ NPs coated with epoxy silane, a new peak at 925 cm^−1^, compatible with the oxirane group in the silane [21], is observed. For WS_2_ NPs coated with vinyl silane, a new peak is observed around 1400 cm^−1^ [19]. For WS_2_ NPs coated with acryloxy silane, a new peak is observed in 1704 cm^−1^, which is related to the vibration of a carbonyl group [19]. For WS_2_ NPs coated with amino silane, a new peak is observed in the range of 1594–1603 cm^−1^ matching the literature value of N-H vibration [20]. Therefore, the IR spectra of the surface-modified WS_2_ NPs attest to the chemical bonding obtained between the silane moieties and the NPs.

**Figure 1:**
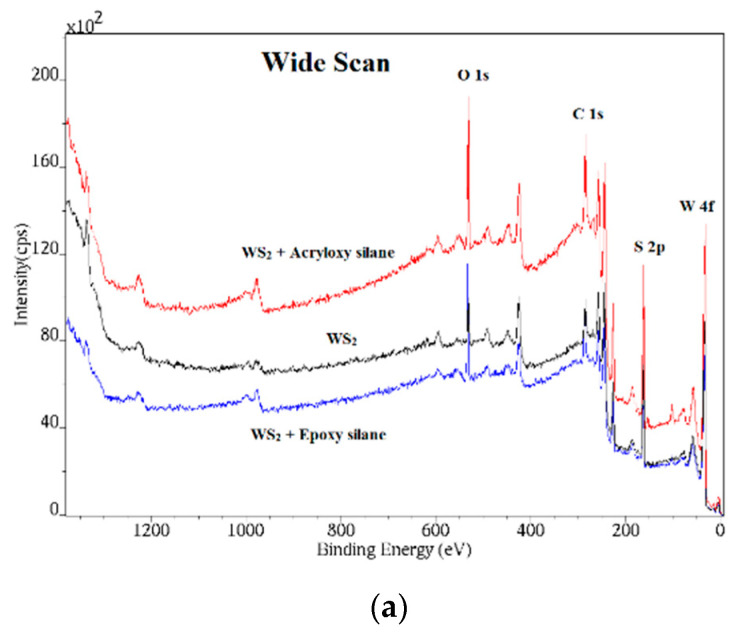

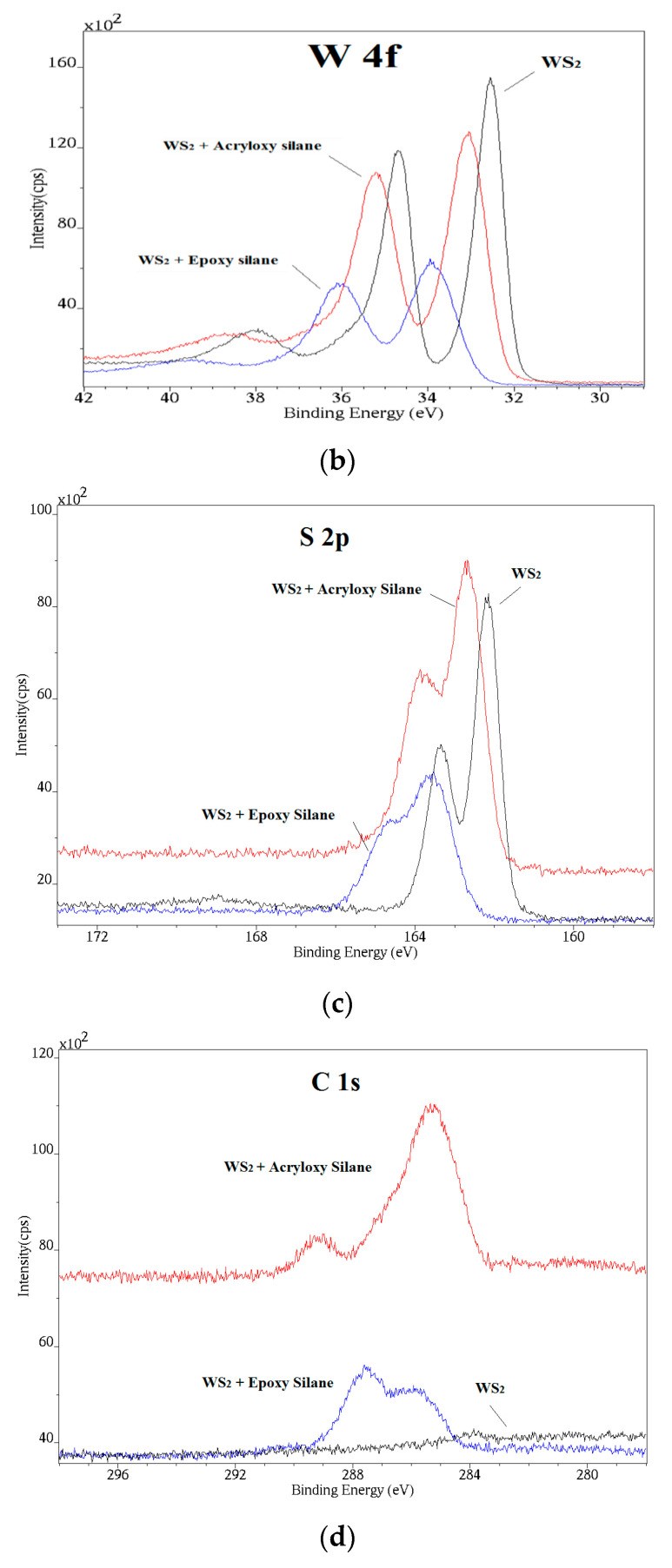
XPS spectra of the silane-treated WS_2_ nanoparticles; pure WS_2_ (black), WS_2_ coated with acryloxy silane (red), and WS_2_ coated with epoxy silane (blue). (**a**) Wide-scan XPS spectra; (**b**) high-resolution W 4f_5/2_; (**c**) S 2p; and (**d**) C 1s spectra.

XPS analysis of the pristine and surface-modified WS_2_ NPs was performed with the purpose of verification and quantification of the covalent bonds that were formed between the WS_2_ NPs and the silane moieties. Although ample evidence from past studies [22] indicates that the IF-WS_2_ NPs are stable in the ambient, minor oxidation of surface defects and adsorption of different moieties from the ambient environment, occurring mostly in the cusps of the folded WS_2_ layers, cannot be fully excluded. Figure 1a shows the wide-scan XPS spectrum of the neat and silane-treated WS_2_ nanoparticles. This spectrum shows that the nanoparticles do not contain any extra impurity other than perhaps adsorbed water molecules or OH moieties [23]. The large oxygen signal visible in the spectrum can be ascribed to the silane moieties adsorbed to the nanoparticles’ surface with minor contribution also from other inadvertently adsorbed oxygenated moieties.

Figure 1b shows a narrow scan of the 4f tungsten line. In the case of relatively conducting NPs surfaces (bare WS_2_ particles), the positive charge induced by the photoelectron ejection is self-compensated by grounding the sample. When the WS_2_ NPs are coated with an insulating (silane) layer, the surface becomes less conducting. Consequently, the charge compensation is not so effective. As a result, a shift of the spectra to positive binding energies is observed. The signal at 38 eV is attributed to the W 5p_3/2_ peak, which is located at slightly more positive energy than the W 4f_5/2_ peak. In general, though, silane moieties are Lewis acids (electron withdrawing from the WS_2_ NPs surface), which means that some of the energy shift of the XPS spectra to higher energies can be attributed to this effect. A thorough discussion on the effect of deposited metal films on the XPS spectra of monolayers of WS_2_ and other transition metal dichalcogenide under UHV conditions can be found in [16,17]. No attempt to quantitatively distinguish between these two contributions has been carried out here. Figure 1c shows the S 2p peaks of the neat IF-WS_2_ NPs and those functionalized with silane moieties. The positive (higher energy) shift of the peaks of the silane-functionalized surfaces can be attributed to the same effect discussed for the W peaks, i.e., the insulating character of the functionalized surface and the electron-withdrawing character of the silane moieties. Figure 1d displays the C 1S spectrum of the different NPs. While the neat IF-WS_2_ NPs show very little carbon content, the silane functionalized surfaces exhibit significant carbon content. The XPS of the surface-modified NPs is also shifted to higher energies compared to the weak 283.8–284.0 eV C 1s peak of carbon on the neat WS_2_ NPs.

The results of the XPS analysis are summarized in Table 1. The overall sulfur-to-tungsten ratio is close to two in all cases, and the deviations are attributed to experimental error or minor surface contamination, which is unavoidable for the NPs processed in ambient conditions. The results clearly reveal that the W-O-Si bonds were indeed formed on the NPs’ surface. Presumably, the oxygen present on the NPs’ surface [24] undergoes a condensation reaction with the silanol groups to form the W-O-Si covalent bonds. For WS_2_ NPs with vinyl coating, no silane bonds could be seen because the coating created a thick insulating film, which prevented direct XPS analysis of the surface-modified specimen. According to the SEM analysis, the thickness of the silane layer on the WS_2_ NPs obtained after the silane treatments ranged from 2.2 to 4.3 nm. For NPs coated with vinyl silane, the thickness of the coating film was the largest, i.e., 4.3 nm, which may explain the impairment of the conductivity of the WS_2_ NPs.

**Figure 2:**
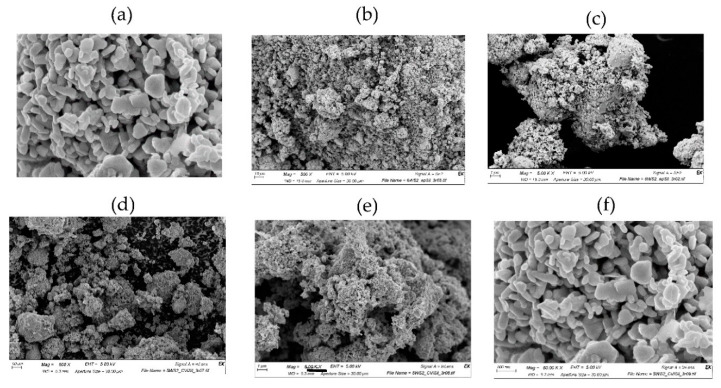
(**a**) WS_2_ neat; (**b**) WS_2_ with epoxy silane ×500; (**c**) WS_2_ with epoxy silane ×5000; (**d**) WS_2_ with vinyl silane ×500; (**e**) WS_2_ with vinyl silane ×5000; (**f**) WS_2_ with vinyl silane ×50k.

**Figure 3:**
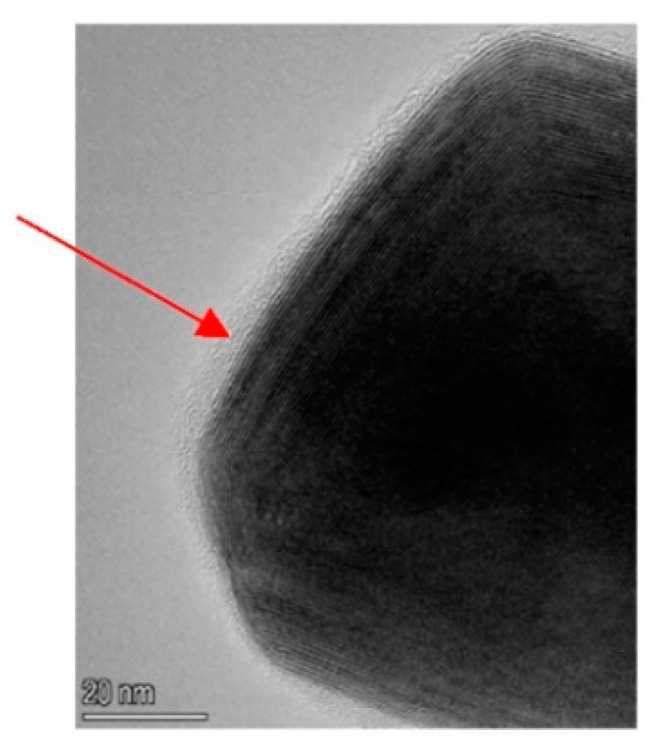
TEM of IF-WS_2_ nanoparticle coated with vinyl silane, (red arrow points to the amorphous coating). Scale bar 20 nm.

The (vinyl) silane-coated WS_2_ NPs were first analyzed by SEM (Figure 2d–f) and subsequently by TEM (Figure 3. The SEM analysis (Figure 2) of the functionalized NPs shows that they remained the same, and their overall shape and size were not affected by the surface treatment. TEM image (Figure 3) indicated that a relatively thick silane layer (4 nm) surrounded the surface-functionalized NPs.

### 3.2. Dispersion of the NPs in the Resin Matrix

The quality of the dispersion and the distribution of the NPs in the nanocomposite is decisive for the nanocomposite behavior. To benefit from the NPs’ properties and achieve optimal performance of the nanocomposite, it is mandatory to break the agglomerates and disperse the NPs thoroughly in the matrix. A preliminary inspection of the quality of the dispersion was obtained by optical microscopy. Visibly, the NPs were well-dispersed in the matrix in all the resins and for all the compositions. However, some agglomerates with an average size of 5 μm were nevertheless visible (limited by the magnification of the optical microscope). A few agglomerates with up to 40 μm in diameter were observed as well.

**Figure 4:**
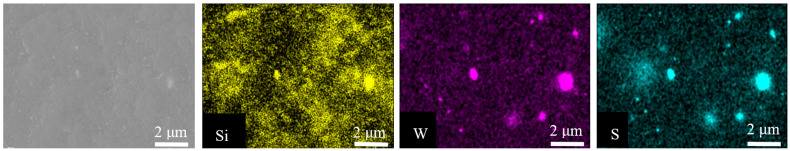
SEM (**left**) and EDS mapping of the EPGnTA sample with 0.5 wt.% WS_2_ NPs coated with vinyl silane. From left to right: secondary electron image; Si (**yellow**); W (**purple**); and S (**cyan**).

Figure 4 shows the EDS analysis for the EPGnTA specimen (#3) with 0.5 wt.% WS_2_ NPs coated with vinyl silane. The Si map (Figure 3a) shows a rather uniform distribution of the silica NPs in the epoxy resin. The silane layer surrounding the NPs was not visible in this analysis for a few reasons. First, the resin was highly insulating, and the silane coating layer on the NPs’ surface was rather thin and invisible on the silica NPs’ background. Furthermore, the overlap between the EDS signal of Si and W (2.2 keV) obscured the silane film. However, there was a good correlation between the sulfur and tungsten signals, which confirms the good quality of the WS_2_ NPs dispersion and distribution in the matrix.

These results indicate that the silica and WS_2_ NPs, notwithstanding their size difference, are properly dispersed in the matrix, making them suitable for well-functioning in the nanocomposite.

### 3.3. Curing Kinetics

An improvement in the curing time was observed for all resins after adding the WS_2_ NPs to the neat resin. In addition, the curing time remained the same for resins with coated and uncoated WS_2_ NPs. It can be concluded, therefore, that the silane coating does not impair the rate of the curing kinetics.

**Figure 5:**
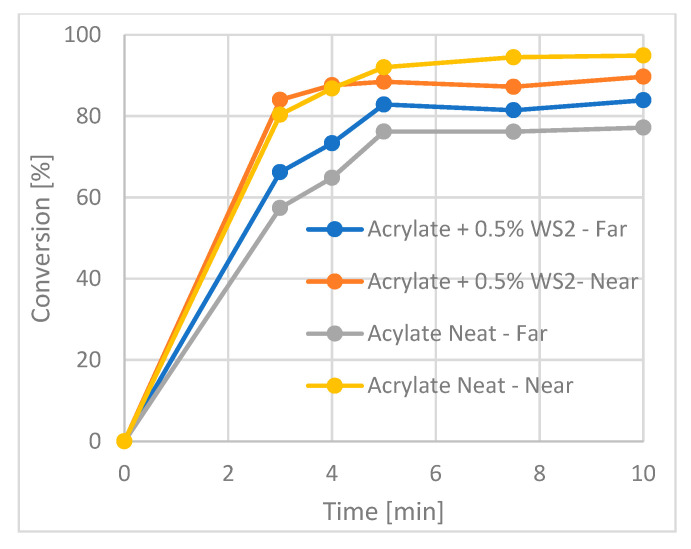
Kinetics of the photocurring of the acrylate sample (#1). The enhanced curing effect of the IF-WS_2_-NPs is demonstrated for the far side.

As for the acrylate (#1) films, longer curing times are required compared to epoxy to achieve a high degree of conversion. Here, radiation times of more than 7.5 min were needed to achieve a degree of conversion of 85%. Figure 5 demonstrates the enhancement in the DC achieved upon the addition of the WS_2_ NPs to the acrylate resin. Both sides of the sample were examined, i.e., in proximity to the UV lamp and on the rear side. Adding the NPs to the acrylate (#1) improved the DC on the far side of the sample, from 77% for the neat polymer up to 84%.

**Figure 6:**
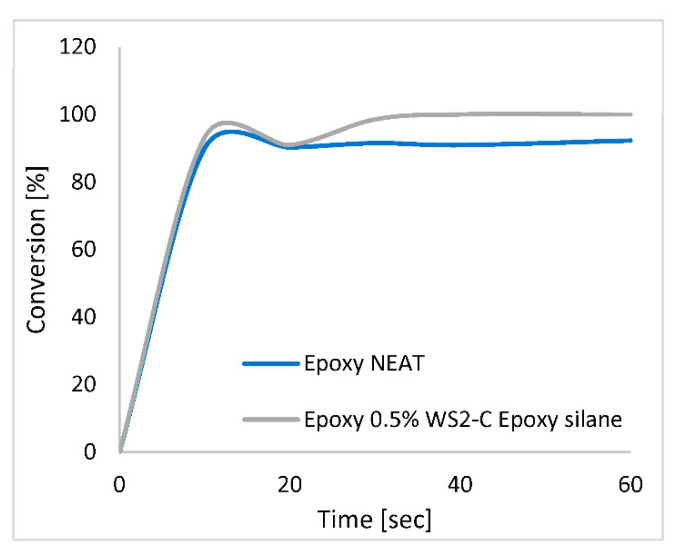
Degree of conversion in the near side of the epoxy.

As for the epoxy resin (#2), it can be seen from Figure 6 that the DC for all compositions reached 92% after 10 s of radiation. However, the epoxy containing the WS_2_ NPs continued the curing beyond this time, while the neat epoxy remained at the same level of conversion, reaching almost 100% conversion after 20 sec of radiation.

The minimum in the conversion observed after about 20 s irradiation time is often observed and could be related to a temporal darkening of the solidifying matrix in the relevant IR range (1620 cm^−1^). Most likely, there is a temporal kinetic mismatch between the diffusion of the monomer molecules and the progressing front of the radical reaction, which leads to a temporal reduction in the rate of reaction. This discrepancy is resolved at later times, and the reaction progresses as expected.

For EPGnTA (#3) (not shown), the DC for all compositions reached 92% after 20 s radiation of the front side of the film. A high DC (92%) was obtained for all the epoxy films containing the WS_2_ NPs, irrespective of their surface functionalization. In the epoxy resin, the photocuring process goes through a cationic reaction, i.e., living polymerization. Therefore, after the initial irradiation had started, the system continued to polymerize and cross-link even after the light had been turned-off and reached a higher DC [25].

### 3.4. Mechanical and Thermal Properties

In our previous publication [25], it was demonstrated that the optimal concentration of the WS_2_ NPs in radiation-induced polymerization of acrylates and epoxies is 0.5 wt.%. Thus, the present study is focused on the effect of 0.5 wt.% of WS_2_ NPs, which were functionalized by silane, on the mechanical and thermal properties of the resultant nanocomposites.

DMA analysis: The glass transition temperature [T_g_] can be evaluated from the maximum tangent *δ* peak [26]. The T_g_ values for all resins and compositions are displayed in Table 2. The pure acrylate (#1) is based on a mixture of IBMA, IBOA, and urethane diacrylate. Here, two T_g_ values were observed at −9 °C and −43 °C, which indicates that the cross-linking was not completed after the photocuring. It is likely that the light induces a very rapid cross-linking of the surface layer, while in the bulk of the film beneath the surface of the acrylate is not fully polymerized. Therefore, the T_g_ peak is either very broad in this case or simply split into two peaks. The peak of T_g_ at −9 °C could be related to methacrylate oligomers that have not been fully cured and remained in the sample after the photocuring. After the addition of the WS_2_ NPs (coated and uncoated), however, only one peak (T_g_) was observed in the photocured acrylate film. These results support the notion of the enhancement effect of the cross-linking (Figure 4), which occurs in the presence of the WS_2_ NPs. The cause of this dip in the DOC curve (See Figure 6) in the presence of the WS_2_ NPs is nevertheless not clear and must be further investigated.

It can be seen from Table 2 that for the treated NPs, the loss modulus is higher than that of a neat acrylate (#1) and acrylate with untreated WS_2_ NPs. The coating of the vinyl silane showed a plasticizer behavior [27]. Indeed, for acrylate film (#1) containing WS_2_ NPs with this coating, the lowest T_g_ (−51 °C) was obtained and the highest loss modulus compared to all other photocured acrylate films. Potentially, the double bond of the vinyl silane and acryloxy silane allows them to participate in the radical reaction, thereby improving the bonding (and mechanical energy transfer) between the acrylate and the WS_2_ NPs. Apparently, the curing of the vinyl bonds interferes with the acrylate structure, producing one with a lower Tg.

For epoxy samples (#2), the T_g_ values increased with the addition of the WS_2_ NPs. The highest T_g_ was obtained for epoxy (#2) with 0.5 wt.% WS_2_ (uncoated).

**Figure 7:**
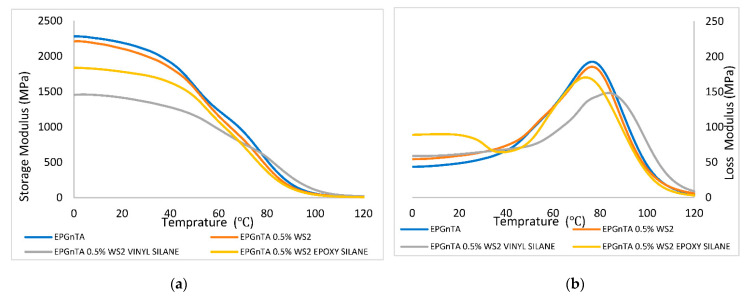
DMA—Storage modulus (**a**) and loss modulus (**b**) of EPGnTA (#3).

The T_g_ values for EPGnTA (#3) are shown in Table 2. In contrast to the acrylate case, here, no significant variations were observed after the addition of the WS_2_ NPs. However, there was a slight increase in the value of T_g_ for the EPGnTA (#3) with WS_2_ NPs after vinyl silane modification. This increase could indicate the formation of chemical bonds between the vinyl group and the polymer. Figure 7 shows the storage modulus and the loss modulus of the neat EPGnTA (#3) and the EPGnTA (#3) containing WS_2_ NPs in different forms. A decrease in storage modulus at low temperatures was observed with the addition of coated WS_2_ NPs. Above a temperature of 80 degrees, all the different EPGnTA (#3) compositions showed the same storage modulus.

Lap shear tests: A clear improvement in the adhesion strength was observed upon the addition of the NPs to the resins. A summary of the results is presented in Table 2. The standard deviations are relatively large. This can be explained by differences in the adhesive thickness, defects in the adhesive layer, such as air bubbles or agglomerations, or uneven surface roughness of the FR4 sheets [25]. Cohesive failure was observed for all the samples.

For acrylate (#1) with 0.5% WS_2_ coated with acryloxy silane, the elongation was improved by 160% in comparison to the neat acrylate. It is assumed that the acryloxy silane has a larger free volume due to the functional group’s extended length. In addition, the energy of breakage was increased compared to the neat acrylate. This result of the acrylate (#1) sample with 0.5 wt.% WS_2_ coated with acryloxy silane correlates well with the increase in T_g_ (obtained by DMA testing) compared to the T_g_ of neat acrylate.

In epoxy (#2) resin, an increase in the adhesion strength was observed upon the addition of the NPs. The highest shear strength was received for epoxy (#2) with 0.5 wt.% WS_2_ NPs coated vinyl silane, i.e., 75% improvement relative to the neat epoxy (#2). A synergistic effect was obtained in the EPGnTA (#3), where a general amelioration of the properties was observed when compared to the pure acrylate (#1) and epoxy (#2) resins. An improvement of 50% in shear strength was obtained for EPGnTA (#3) with 0.5% uncoated WS_2_ NPs compared to the neat EPGnTA (#3). The shear strength obtained for the two epoxies resins (#2, #3) with WS_2_ NPs coated with epoxy silane was about the same (~13.5 MPa); however, the energy for breaking of EPGnTA (#3) was higher than that of epoxy (#2).

Impact tests: As expected, compared to the neat EPGnTA (#3), the impact fracture energy required for nanomaterials containing the NPs is larger. Sample #3, containing the WS_2_ NPs treated with vinyl silane, showed a higher improvement of 73% in impact resistance and a small standard deviation (Table 2) compared with the neat resin. These results indicate that the vinyl silane-coated NPs do bind to the resin and increase its stiffness. These results are also consistent with the increase in T_g_ obtained for the same composition in the DMA test.

**Figure 8:**
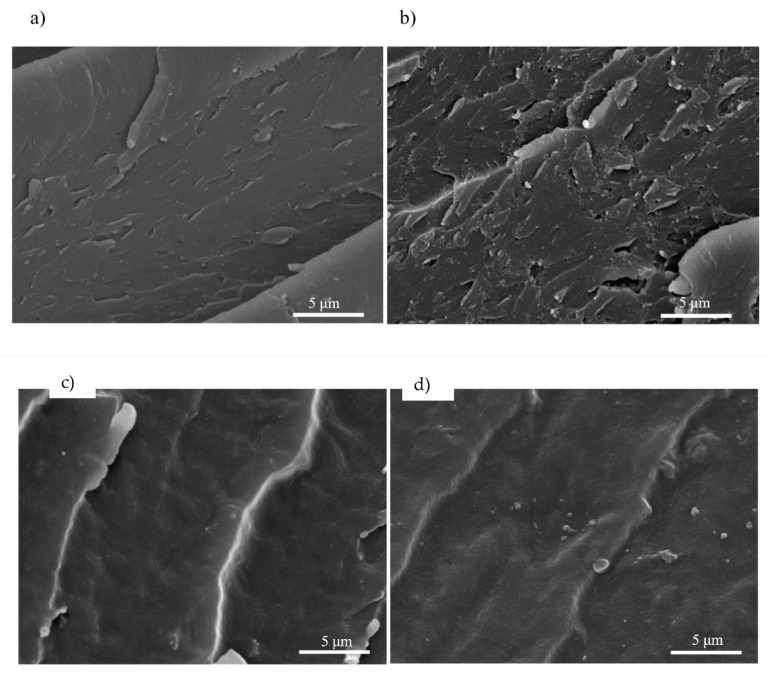

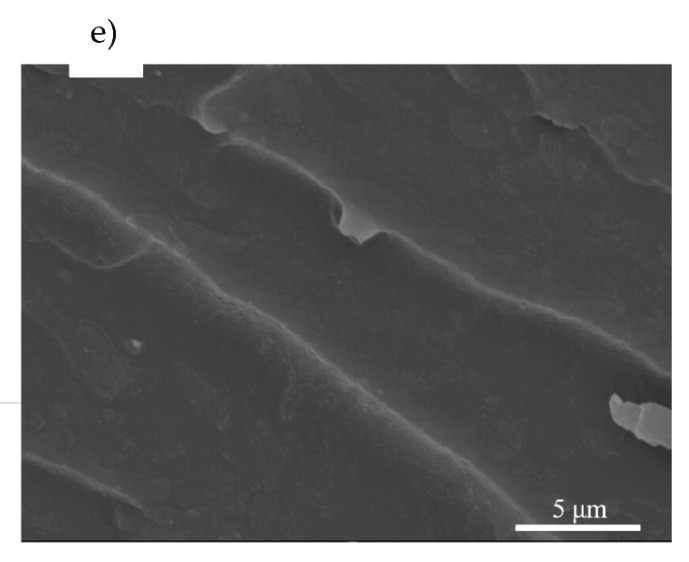
SEM analysis of fractured surface after impact test: (**a**) EPGnTA neat; (**b**) EPGnTA with 0.5 wt.% WS_2_—coated acryloxy silane; (**c**) EPGnTA with 0.5 wt.% WS_2_—coated by vinyl silane; (**d**) EPGnTA with 0.5 wt.% WS_2_—coated by epoxy silane; (**e**) EPGnTA with 0.5 wt.% WS_2_.

The morphology of the fractured surface of the EPGnTA (#3) specimen was examined by SEM after the impact test (see Figure 8). A difference can be seen in the morphology of neat EPGnTA (#3) in comparison to EPGnTA (#3) nanocomposites with neat and coated WS_2_ NPs. Furthermore, a comparison of the neat EPGnTA (#3) and the EPGnTA containing WS_2_ NPs coated with acryloxy silane indicates that the latter specimen showed the roughest surface out of all tested samples. Finally, SEM images of fractured surfaces of EPGnTA (#3) with uncoated WS_2_ NPs and WS_2_ NPs coated with epoxy silane and vinyl silane exhibited a smoother surface morphology with straight lines and clear shear bands. Increased fracture toughness of the nanocomposites containing the NPs can possibly be attributed to the formation of cavities around the NPs.

## 4. Conclusions

Photocuring of epoxy and acrylate resins containing small amounts of pristine and silane surface-functionalized WS_2_ NPs were studied and interrelated to its direct conversion (DC) and its mechanical properties. Very good dispersion of the NPs in the resins was achieved by combining sonication and vortex mixing. Clear evidence in support of the surface functionalization was obtained from TEM and XPS analyses. The adhesion strength increased for all resins after the addition of surface silane-modified NPs. The standard deviations obtained in the impact and shear adhesion tests for the resins containing modified NPs were lower compared to the neat resins and resins filled with unmodified WS_2_ NPs. This observation indicates a more uniform distribution of the NPs in the resin matrix. A synergistic effect was obtained in the EPGnTA (#3), where a general amelioration of the mechanical properties was observed when compared to the pure acrylate (#1) and pure epoxy (#2) resins.

A previous study has shown that adding these NPs at a concentration of 0.5 wt.% is the ultimate loading for improving the resin’s mechanical properties. This study supports these earlier findings not solely for the neat NPs but also for those functionalized with silane moieties. This investigation is the first one to study the effect of silane treatments of WS_2_ in radiation-induced curing of acrylates and epoxies, and it opens new vistas for the photocuring of resins and polymers in general.

## Figures and Tables

**Table 1:** Atomic concentration ratios and (average) thickness of the silane coating on the WS_2_ NPs’ surface derived from the XPS analysis.

Sample/ Ration	S_tot_/w_tot_	S_red_/w_red_	W_osi_/w_tot_	N_tot_/Si	N_red_/Si	Silane Layer, À (Max)	Silane + Top Impurities, À (Max)
Bare	1.88	2.05	-			-	2.9
Coated—vinyl silane	2.13	-	-			42.7	42.9
Coated—epoxy silane	2.14	2.18	0.06			39.4	39.7
Coated—Amino silane	2.12	2.09	0.1	0.95	0.97	21.7	22.1
Coated—Acrloxy silane	2.16	2.25	0.06			35.8	36.0

**Table 2:** DMA, lap shear, tensile and impact result of EPGnTA, epoxy, and acrylate.

	WS_2_ wt.%	Surface Modification by Silane	Tg (°C)	Storage Modulus at 20 °C (MPa)	Storage Modulus at 50 °C (MPa)	Loss Modulus at 20 °C (MPa)	Loss Modulus at 50 °C (MPa)	Shear Strength (MPa)	Energy to Break (J)	Impact (KJ/m^2^)
EPGnTA	0	-	100	2191	1579	49	94	11.9 ± 1.3	6.2 ± 0.9	1.1 ± 0.2
	0.5	Uncoated	98	2017	1546	59	95	17.7 ± 3.6	6.8 ± 2.8	1.7 ± 0.7
	0.5	Coated epoxy silane	99	1785	1455	89	77	13.9 ± 1.4	13.5 ± 2.9	1.7 ± 0.3
	0.5	Coated vinyl silane	105	1414	1162	61	73	13.9 ± 0.7	17.1 ± 3.6	1.8 ± 0.1
	0.5	Coated acryloxy silane	113	890	1213	19	41	13.8 ± 3.3	6.6 ± 2.2	1.2 ± 0.3
	0.5	Coated amino silane	111	1418	784	30	27	15.2 ± 1.6	17.2 ± 1.2	1.1 ± 0.4
Epoxy	0	-	108	1369	916	37	77	8.6 ± 0.4	3.1 ± 0.2	
	0.5	Uncoated	127	731	608	20	31	12.1 ± 1.6	5.9 ± 1.8	
	0.5	Coated epoxy silane	115	708	573	21	30	10.9 ± 0.4	4.7 ± 0.2	
	0.5	Coated vinyl silane	119	1210	957	31	43	13.5 ± 1.4	6.5 ± 1.5	
Acrylate	0	-	−9, −43	258	-	30	-	4 ± 0.1	1.6 ± 0.7	
	0.5	Uncoated	−34	256	135	30	22	4.7 ± 0.3	4.1 ± 1.0	
	0.5	Coated acryloxy silane	−31	577	299	57	47	3.1 ± 1.1	1.0 ± 0.4	
	0.5	Coated vinyl silane	−51	1781	1376	69	88	4.2 ± 0.5	5.3 ± 2.0	

## Data Availability

The data that support the findings of this study are available on request from the corresponding author.
